# Correction to: Laparoscopic rectal resection without epidural catheters—does it work?

**DOI:** 10.1007/s00384-024-04612-z

**Published:** 2024-03-23

**Authors:** M. El-Ahmar, F. Koch, A. Köhler, L. Moikow, M. Ristig, J.-P. Ritz

**Affiliations:** 1 Department of General and Visceral Surgery, Helios Kliniken Schwerin, 19055 Schwerin, Germany; 2 Department of General and Visceral Surgery, Charité Universitätsmedizin Berlin Campus Benjamin Franklin, Hindenburgdamm 30, 12203 Berlin, Germany; 3 Department of Anesthesiology, Helios Kliniken Schwerin, 19055 Schwerin, Germany

**Correction to: International Journal of Colorectal Disease (2022) 37:2031-2040** 10.1007/s00384-022-04242-3

In this article additional affiliation has been added to the first author 'M. El-Ahmar' as 'Department of General and Visceral Surgery, Charité Universitätsmedizin Berlin Campus Benjamin Franklin, Hindenburgdamm 30, 12203 Berlin, Germany'.

